# Genetic variants in eleven central and peripheral chemoreceptor genes in sudden infant death syndrome

**DOI:** 10.1038/s41390-021-01899-4

**Published:** 2022-02-01

**Authors:** Jacqueline Neubauer, Anna-Lena Forst, Richard Warth, Christian Peter Both, Cordula Haas, Jörg Thomas

**Affiliations:** 1 Zurich Institute of Forensic Medicine, University of Zurich, Zurich, Switzerland; 2 Medical Cell Biology, Institute of Physiology, University of Regensburg, Regensburg, Germany; 3 Department of Anesthesiology, University Children’s Hospital Zurich, Zurich, Switzerland

## Abstract

**Background:**

Sudden infant death syndrome (SIDS) is still one of the leading causes of postnatal infant death in developed countries. The occurrence of SIDS is described by a multifactorial etiology that involves the respiratory control system including chemoreception. It is still unclear whether genetic variants in genes involved in respiratory chemoreception might play a role in SIDS.

**Methods:**

The exome data of 155 SIDS cases were screened for variants within 11 genes described in chemoreception. Pathogenicity of variants was assigned based on the assessment of variant types and in silico protein predictions according to the current recommendations of the American College of Medical Genetics and Genomics.

**Results:**

Potential pathogenic variants in genes encoding proteins involved in respiratory chemoreception could be identified in 5 (3%) SIDS cases. Two of the variants (R137S/A188S) were found in the *KNCJ16* gene, which encodes for the potassium channel Kir5.1, presumably involved in central chemoreception. Electrophysiologic analysis of these *KCNJ16* variants revealed a loss-of-function for the R137S variant but no obvious impairment for the A188S variant.

**Conclusions:**

Genetic variants in genes involved in respiratory chemoreception may be a risk factor in a fraction of SIDS cases and may thereby contribute to the multifactorial etiology of SIDS.

**Impact:**

- What is the key message of your article?

Gene variants encoding proteins involved in respiratory chemoreception may play a role in a minority of SIDS cases.

- What does it add to the existing literature?

Although impaired respiratory chemoreception has been suggested as an important risk factor for SIDS, genetic variants in single genes seem to play a minor role.

- What is the impact?

This study supports previous findings, which indicate that genetic variants in single genes involved in respiratory control do not have a dominant role in SIDS.

## Introduction

Sudden infant death syndrome (SIDS) is defined as the sudden and unexpected death of an infant younger than 1 year of age, with the onset of the fatal episode apparently occurring during sleep.^1^ Although the incidence of SIDS has decreased over the last decades, SIDS is still one of the leading causes of post-neonatal infant death in developed countries with a prevalence between 0.1 and 0.8 deaths per 1000 live births.^2^ The occurrence of SIDS is described by a complex multifactorial etiology involving (i) a vulnerable infant, (ii) a critical period of development in homeostatic control, and (iii) exogenous stressors.^3^ While many of the exogenous stress factors, such as the prone sleeping position, overheating, or maternal smoking during pregnancy, are well-known risk factors, the genetic pathogenesis leading to the sudden death of an infant remains poorly understood.^4^ Numerous case-control studies have focused on genetic determinants predisposing an infant to an increased vulnerability by investigating several pathophysiological mechanisms, such as metabolic diseases, immune system dysfunction, or central nervous and brain development.^5^ In addition, massive parallel sequencing (MPS) has provided a comprehensive and time-efficient sequencing strategy to identify rare, likely disease-causing variants associated with complex diseases in sudden unexplained death cases.^6,7^ Whole-exome or targeted gene panel sequencing in several SIDS cohorts mainly focused on cardiac diseases and identified likely pathogenic variants within 200 cardiac genes in up to 30% of SIDS cases.^8–11^

**Fig. 1:**
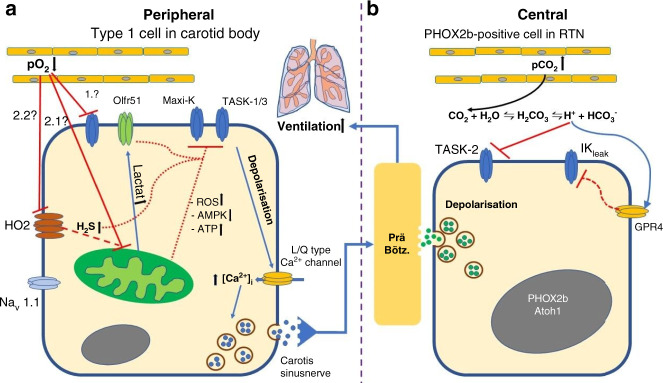
Cell models of peripheral and central chemoreception. **a** Proposed mechanism of oxygen-sensing in the carotid body. An inhibition of potassium channels (TASK-1/3, maxi-K^+^) in type I cells induces a depolarization of the cell membrane with activation voltage-sensitive Ca^2+^ influx. Two main different theories exist, how the inhibition of membrane K^+^ channels takes place: (1.) Hypoxia induces direct inhibition of the K^+^ channels or (2.1/2.2) a rise of metabolic factors, like ROS, in the mitochondria inhibits the K^+^ channels. *pO*_*2*_ partial pressure oxygen; *HO*_*2*_ heme-oxygenase 2; *H*_*2*_*S* hydrogen sulfide; *Nav1.1* voltage-gated sodium channel type I; *ROS* reactive oxygen species; *L/Q-type Ca*^*2+*^ *channel* voltage-gated calcium channel; *TASK-1 + 3* Twik (Tandem of P-domains in a Weakly Inward rectifying K^+^ channel)-related acid-sensitive potassium channels; *Maxi-K*^+^ large conductance Ca^2+^-activated K^+^ channel; *Olfr51* Olfactory receptor family 51, *Glu* glutamate. **b** The currently accepted model of CO_2_ measurement in the retrotrapezoid nucleus (RTN) of the brainstem. Increase of partial CO_2_ pressure (pCO_2_) and thereby acidification of the cerebroid fluid directly inhibits TASK-2 and directly activates GPR4. The inhibition of TASK-2 and other K^+^ channels (IK_leak_) by GPR4 depolarize cell membrane of the PHOX2B positive neurons and induces thereby neurosecretion. *ATP* adenosine-tri-phosphate; *pCO*_*2*_ partial pressure of carbon dioxide; *TASK-2* Twik (Tandem of P-domains in a Weakly Inward rectifying K^+^ channel)-related acid-sensitive potassium channels; *GPR4* G-protein-coupled receptor 4; *IK*_*leak*_ leak potassium channels; *CPG* central pattern generator; *PHOX2B* paired-like homeobox 2B; *ATOH1* atonal homolog 1.

An interesting pathway in the pathogenic mechanism of SIDS is the ventilator control system that includes central respiratory rhythmogenesis, central and peripheral chemoreception and modulation in the central nervous system.^12^ Although ventilator control development in the central brainstem begins early in gestation, respiration in the newborn is immature and instable, and nearly any form of stress, including simple alterations in body temperature, may cause apnea.^13,14^ Respiratory chemoreception is also immature at birth, particularly the ability to sense hypoxia, which has to be “learned” in the first month of life.^15^ This is achieved by fundamental changes in the carotid bodies (CB), the main peripheral respiratory chemoreceptors, which are located in the arch of the carotid arteries.^16^ In addition, the vulnerable phase of carotid body development takes place during the same period where the risk for SIDS is at its’ highest.^17^ In chemoreceptive cells, membrane proteins, such as ion channels and receptor proteins, are indispensable for the measurement of changes to the partial pressure of oxygen (pO_2_), carbon dioxide (pCO_2_) or hydrogen ions (pH) in the arterial blood or brain tissue to either activate or inhibit the central pattern generator (CPG).^18^ The chemo-sensitive cells in the carotid body are the so-called type 1 cells (glomus cells), which are mitochondria-rich cells expressing numerous different ion channels, including TASK (Twik-associated acid-sensitive K^+^ channel) channels (TASK-1/TASK-3), calcium- and voltage-activated K^+^ channels (Maxi-K^+^), voltage-gated Na^+^ channels (Na_v_), and L- and N-type Ca^2+^ channels.^19^ Despite intensive research, the exact function of these ion channels in the chemoreception of the CB is still not fully understood. Although is not definitively established where and how oxygen-sensing takes place in the glomus cells, recent studies suggested that mitochondrial changes are sensed by membrane channels.^20^ An overview of the proposed mechanisms involved in hypoxia-sensing in the CB is depicted in Fig. 1a.

By contrast, the mechanism of central respiratory chemoreception has become more clear. The retrotrapezoid nucleus (RTN), the most important central respiratory chemoreceptive area located in the ventrolateral medullary surface under the facial motor nucleus, was identified in 1989.^21^ The RTN activates respiration under hypercapnia and/or acidotic conditions and it is thought to modify in addition the hypoxia answer.^22^ Nevertheless, the RTN is thought to provide the most important chemosensory drive to stimulate respiration during CO_2_ increases or acidosis.^23^ A cluster of glutamatergic neurons in the RTN expressing the transcription factors *PHOX2B* (Paired-like homeobox 2b) and *ATOH1* (atonal homolog 1) are responsible for this chemosensory function.^23^ Both transcription factors are suggested to be very important in the normal development of these neurons and their ability to sense CO_2_ changes.^24^ Individuals who carry specific mutations in the *PHOX2B* gene, like the polyalanine repeat in exon 3, are known to develop central congenital hypoventilation syndrome (CCHS).^25^ In this respect, variants in the *PHOX2B* gene are thought to play a role in SIDS,^26^ but the relevance of these results is still under debate.^27^ Recent studies have identified two important proteins essential for CO_2_ chemoreception in *PHOX2B* positive neurons in the RTN: the background potassium channel TASK-2 and the protein-coupled receptor 4 (GPR4).^22,28^ Absence of either TASK-2 or GPR4 impairs the central respiratory CO_2_ chemoreflex and the deletion of both proteins virtually eliminates it.^28^ The currently accepted model of central chemoreception in the RTN is shown in Fig. 1b. The locus coeruleus is also thought to be involved in central CO_2_ chemoreception and the transcription factor Methyl-CpG binding protein 2 (MECP2) and inwardly rectifying K^+^ channel 5.1 (Kir5.1/*KCNJ16*) are suggested to be important for appropriate CO_2_ responses.^29,30^

Laer et al. have examined a SIDS cohort for single nucleotide polymorphisms (SNPs) in genes encoding some of the key modulators of respiratory control.^31^ They focused on known SNPs in different transmitter systems (41 genes) associated with respiratory control in a large SIDS cohort (366 cases).^31^ In certain subgroups of SIDS cases, they showed a significant association for two polymorphisms, one in the opioid receptor mu1 (*OPRM1*, subgroup: death occurring during autumn) and one in the sulfotransferase 1A1 (*SULT1A1*, subgroup: death occurring during summer). The authors concluded that it is very unlikely that one of the investigated polymorphisms in these genes associated with respiratory control exerts a strong effect on the predisposition towards SIDS.^31^ However, they did not specifically search for SNPs in genes encoding proteins involved in central or peripheral chemoreception. This study, therefore, aims to analyze genetic variants in 11 genes involved in the central and peripheral chemoreception by analyzing the exome data of 155 SIDS cases.

## Material and methods

### SIDS study population

The study population consisted of 155 SIDS cases collected between 1985 and 2014 at the Zurich Institute of Forensic Medicine (ZIFM), Switzerland. Most cases were examined by the same forensic pathologist, ensuring a high level of consistency in autopsy procedures and case reporting. The classification of SIDS cases has always been performed according to the latest accepted international definitions of SIDS, including a complete autopsy, review of the circumstances of death, and inspection of the clinical history.^32^ The median age at death of the 155 SIDS infants was 17.4 ± 10.67 weeks (range 0.6–48.1 weeks) and 62.2% were boys (94 males/61 females). All of the SIDS infants were of European ancestry, most of them Swiss. Ethical approval for this study was provided by the local ethics committee (KEK-ZH-No. 2013-0086), and the study was conducted in full conformance with Swiss laws and regulations. Family members were not available for co-segregation analysis.

### Exome sequencing

The exome sequencing procedure of the 155 SIDS cases has been described in detail in Neubauer et al.^11^ In brief, genomic DNA of the SIDS infants was obtained from tissues (kidney or brain) stored in alcohol or from alcohol-fixed and paraffin-embedded tissue blocks. DNA extraction was performed using the QIAamp DNA Mini Kit (Qiagen, Hombrechtikon, Switzerland) according to the manufacturer’s protocol. The SureSelectXT All Exon V5 + UTR kit (Agilent Technologies AG, Basel, Switzerland) was used for DNA library preparation and the sequencing was done on the Illumina HiSeq2500 platform (Illumina Inc., San Diego). Sequences were aligned to the reference genome (GRCh37/hg19) using BWA^33^ and samples were required to have a least 80% of the exome covered at ≥20× read depth. Variant discovery was performed by means of Genome Analysis Toolkit (GATK),^34^ following the GATK best practice workflow.^35^

### Exome data analysis

The exome data of the 155 SIDS cases were filtered for variants within 11 genes described in central and peripheral chemoreception (Table 1a and b and Supplementary Table S1). Annotation of the variants was performed with the Software Alamut Batch version 1.9 (Interactive Biosoftware, Rouen, France). Output results were reported in an excel-sheet for data analysis. Variants were filtered according to our in-house filter strategy.^11^ Filter criteria were (1) a global minor allele frequency value (MAF) of less than or equal to 0.001 derived from the Genome Aggregation Database (gnomAD, https://gnomad.broadinstitute.org/),^36^ (2) focus on exonic and splice site variants, and (3) the exclusion of synonymous variants. Alamut Visual Version 2.1.0 (Interactive Biosoftware) and Integrative Genomics Viewer version 2.4 (Broad Institute, Massachusetts) were used to visualize coverage of variants and to review the conservation of the variants across a variety of species. Pathogenicity of variants was assigned based on the assessment of variant types (null-variants, frameshift variants, splice site variants, or missense variants) and in silico protein predictions according to the recommendations of the American College of Medical Genetics and Genomics standards and guidelines (ACMG) for the interpretation of sequence variants.^37^ Co-segregation and functional analyses would have been required to classify a variant as pathogenic; therefore, all sequence alterations were labeled as variants of uncertain significance in this study.

**Table 1:** Genes encoding for proteins involved in (a) peripheral respiratory chemoreception of the carotid body (CB) and in (b) central respiratory chemoreception. Variants in these genes may restrict the chemoreception in the CB, retrotrapezoid nucleus (RTB) or locus coeruleus (LC), but may also alter the function of other organs.

Genes	pLI	Description	Function	Other disorders	Source
(a) Peripheral respiratory chemoreception					
*KCNK9*	**0.9**	2P-K-channel, member 9 (TASK-3)	O_2_/CO_2_/H^+^ in the CB	Epilepsy	F: Kim (2009)^16^
*KCNK3*	**0.9**	2P-K-channel, member 3 (TASK-1)	O_2_/CO_2_/H^+^ in the CB	Primary pulmonary hypertension	F: Kim (2009)^16^ OD: Ma (2013)^56^
*OR51E2*	0	Olfactory receptor family 51 subfamily E member 2	O_2_/CO_2_/H^+^ in the CB		F: Chang (2015)^44^
*KCNMA1*	**1**	Calcium-activated K^+^ channel subfamily M alpha 1 (Maxi-K^+^)	Peripheral chemoreception	Generalized epilepsy, cerebral atrophy, developmental delay	F: Gomez-Nino (2009)^48^ OD: Du W (2005)^49^
*NDUFS2*	0	NAD: ubiquinone oxidoreductase core subunit S2	Peripheral chemoreception	Mitochondrial complex I deficiency, leukodystrophy	F: Fernandez (2015)^20^ OD: Schuelke (1999)^59^
(b) Central respiratory chemoreception					
*KCNK5*	0.33	2P-K-channel, member 5 (TASK-2)	CO_2_ chemoreception RTN	Renal acidosis	F: Gestreau (2010)^22^ OD: Warth (2004)^57^
*GPR4*	0.1	G-protein-coupled receptor 4 (GPR4)	CO_2_ chemoreception RTN	Intestinal inflammation	F: Kumar (2015)^28^ OD: Wang (2018)^58^
*KCNJ16*	0	Inwardly rectifying K^+^ channel 5.1 (Kir5.1)	CO_2_ chemoreception LC	Renal acidosis	F: D’Adamo (2011)^30^ OD: Puissant (2019)^54^
*PHOX2B*	**0.94**	Paired-like homeobox 2B (PHOX2B)	CO_2_ chemoreception RTN brain development	CCHS	F: Amiel (2003)^25^
*ATOH1*	0.02	Atonal homolog 1 (ATOH1)	CO_2_ chemoreception RTN brain development		F: Ruffault (2015)^24^
*MECP2*	**0.89**	Methyl-CpG binding protein 2 (MECP2)	CO_2_ chemoreception in LC brain development	Rett-syndrome (epileptic seizures)	F: Zhang (2010)^29^

*TASK-1 + 3* Twik (Tandem of P-domains in a weakly inward rectifying K^+^ channel)-related acid-sensitive potassium channels.

*RTN* retrotrapezoid nucleus, *LC* locus coeruleus, *CCHS* central congenital hypoventilation syndrome, *OD* other disorder, *F* function, *pLI* Probability of being loss-of-function intolerance from the Genome Aggregation Database (gnomAD) browser.^36^ pLI ≥ 0.9 extremely loss-of-function intolerant genes (printed in bold).

### Variant confirmation

Our minimum threshold to interpret the exome sequencing data was defined as 20× coverage.^38^ Variants with < 50× bidirectional coverage and/or an alternate allele frequency ratio < 0.4, and potential disease-causing variants not reported in the mentioned databases were in addition confirmed by conventional Sanger sequencing (Supplementary Fig. S1).

### Electrophysiological analysis of Kir5.1 (KCNJ16) variants

Experiments were essentially performed as described previously.^39^ Human embryonic kidney 293T (HEK293T) cells were transiently transfected with 0.1 µg hKCNJ10-pIRES_CD8 plasmid. For hKCNJ16-HA-pIRES_CD8 or mutant hKCNJ16 variants containing plasmids, always 0.9 µg were used for transfection or co-transfection with KCNJ10. The stoichiometric ratio of 1:10 was used to prevent homomeric KCNJ10 channel assembly. Measurements were performed one day after transfection of cells. Whole-cell patch-clamp recordings were performed using an EPC-10 amplifier (HEKA) and the PatchMaster software (HEKA). The patch pipette solution was composed of 95 mM K-gluconate, 30 mM KCl, 4.8 mM Na_2_HPO_4_, 1.2 mM NaH_2_PO_4_, 5 mM Glucose, 1 mM EGTA, 2 mM Na-ATP, 2.38 mM MgCl_2_ and 0.726 mM CaCl_2_ (pH 7.2). The standard bath solution for whole-cell experiments contained 145 mM NaCl, 5 mM Hepes, 1.6 mM K_2_HPO_4_, 0.4 mM KH_2_PO_4_, 5 mM Glucose, 1 mM MgCl_2_ and 1.3 mM CaCl_2_ (pH 7.4). The liquid junction potential was 10 mV and was corrected by the PatchMaster software. High K^+^ bath solution contained 5.0 mM Hepes 5.0, 98.6 mM NaCl, 46.4 mM KCl, 1.6 mM K_2_HPO_4_, 0.4 mM KH_2_PO_4_, 5 mM Glucose, 1 mM MgCl_2_ and 1.3 mM CaCl_2_ (pH 7.4). All measurements were carried out at room temperature. Current-voltage relationships were obtained from a voltage stair protocol from −120 to 30 mV in 30 mV increments (duration each step 500 ms). After each stair, a current clamp protocol was executed that clamped the cells to zero current.

### Statistics

Analysis for parametric and nonparametric data was performed by Shapiro-Wilk test. Descriptive statistics with differences between groups are shown as mean with standard deviation for parametric data and as median with 95% confidence interval (95%CI) for nonparametric data. For statistical significance, Mann-Whitney or one-way ANOVA using the Origin2019 software (OriginLab) were performed for nonparametric and parametric data respectively, with *p* < 0.05 considered to be statistically significant. Tukey post-hoc was used to assess significances between groups.

## Results

In total, exons of 11 genes encoding for different proteins involved in central or peripheral respiratory chemoreception were analyzed for variants in our SIDS cohort of 155 cases (Table 1a and b and Supplementary Table S1). Overall, a coverage of ≥20 reads was achieved in 72.3% of the bases and the average on-target coverage was 90.2% at ≥20 reads. The 11 genes of interest had a minimum mean exonic coverage of 29.98 ± 15.01 and a maximum mean exonic coverage of 197.39 ± 87.13 for the 155 SIDS cases (Supplementary Fig. S1). Within these genes, an average of 41.58 ± 11.35 variants per case were obtained for further evaluation. These variants were filtered according to the above-mentioned filter strategies and the final candidates were manually assessed and evaluated according to our scoring scheme. Sanger sequencing confirmation was performed for one variant, which had a coverage below 50× bidirectional reads (Supplementary Fig. S2).

Five potential pathogenic variants in the coding regions of genes involved in respiratory chemoreception were identified in 5 (3%) SIDS cases (Table 2). Two heterozygous variants were found in important components of peripheral chemoreception in the carotid body, such as the coding region for the olfactory receptor (*OR51E2*; rs777767053; c.535G>T; V179F) and in the coding region of the calcium-activated K^+^ channel *(KCNMA1*; new variant; c.3517A>G; S1173G). Genetic variants of proteins involved in central chemoreception were found in three SIDS cases, one in the transcription factor paired-like homeobox 2B (*PHOX2B*; new variant; c.353C>T; A118V), and two in the inwardly rectifying K^+^ channel 5.1 (*KCNJ16*; new variant; c.667G>T; A188S and rs766250689; c.409C>A; R137S). Four of the 5 SIDS cases were males and the median age at death of all 5 SIDS cases was 10 weeks [95% confidence interval (95%CI): 6.4–15] compared to 17 weeks [95% CI: 11-20] in the SIDS cases with potential cardiovascular genetic diseases (*n* = 28; *p* = 0.047).^11^

**Table 2:** **Potentially pathogenic variants identifed in the SIDS cohort.** In three SIDS cases potentially pathogenic variants were found in genes of central chemoreception (*PHOX2B/KCNJ16*) and in two SIDS cases in genes of peripheral chemoreception (*OR51E2*/*KCMA1*).

Case	Gender	Sleeping position	Preterm birth (<37)	Age (months)	rs-Nr.	Gene	HGVS genomic RefSeq-Nr.	HGVS RefSeq-Nr.	Coding effect	cDNA	Protein change	gnomAD ALLMAF	Grantham distance score	AGVGD	SIFT	MAPP	MutationTaster	Polyphen2	Coverage	Heterozygous allele frequency ratio	Pathogenicity
SIDS025	Female	Prone	No	3	–	*PHOX2B*	CHR4(GRCh37):g.41749442G>A	NM_003924.3	Missense	c.353C>T	A118V	0.000000	64	C65	Deleterious	Bad	Disease-causing	Probably damaging	50	0.50	Uncertain significance
SIDS091	Male	Prone	No	2	–	*KCNJ16*	Chr17(GRCh37):g.68128790G>T	NM_001270422.1	Missense	c.667G>T	A188S	0.000000	99	C65	Deleterious	NA	Polymorphism	Probably damaging	37	0.44	Uncertain significance
SIDS131	Male	Prone	No	2	rs777767053	*OR51E2*	Chr11(GRCh37):g.4703407C>A	NM_030774.3	Missense	c.535G>T	V179F	0.000032	50	C45	Deleterious	Bad	Polymorphism	Possibly damaging	78	0.42	Uncertain significance
SIDS107	Male	NA	Yes	4	rs766250689	*KCNJ16*	Chr17(GRCh37):g.68128637C>A	NM_001270422.1	Missense	c.409C>A	R137S	0.000000	110	C65	Deleterious	NA	Disease-causing	Probably damaging	94	0.45	Uncertain significance
SIDS192	Male	Prone	Yes	3	–	*KCNMA1*	Chr10(GRCh37):g.78647137T>C	NM_001322837.1	Missense	c.3517A>G	S1173G	0.000011	56	C0	Deleterious	NA	Disease-causing	Probably damaging	45	0.30	Uncertain significance

*AGVGD* align Grantham variation and Grantham deviation, *gnomAD* MAF in all populations based on the genome aggregation database, *MAF* minor allele frequency, *MAPP* multivariate analysis of protein polymorphism prediction, *NA* not available, *SIFT* sorting intolerant from tolerant prediction.

**Fig. 2:**
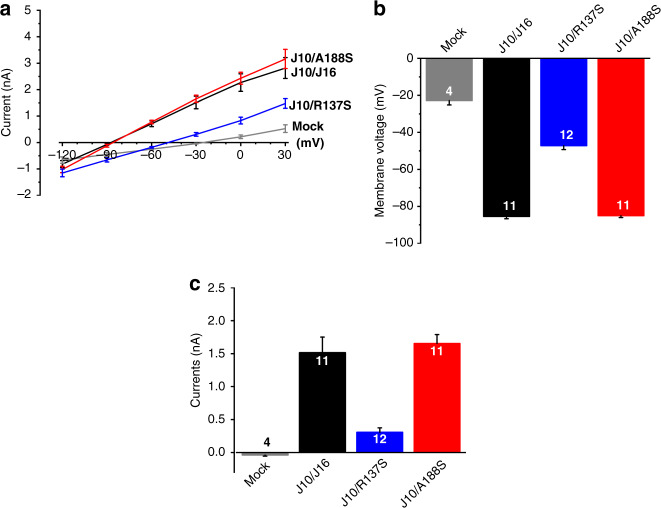
Electrophysiological characterization of KCNJ16 variants. **a** Current–voltage curves of human embryonic kidney cells (HEK293T) either non-transfected (mock; gray line) or co-transfected with KCNJ10 (J10) and KCNJ16WT (J16, black line) or mutant KCNJ16 (R137S, blue line; A188S, red line). The variant R137S seemed non-functional, whereas A188S was fully functional. Data are presented as mean values ± SEM. **b** Resting membrane potential in HEK293T cells: KCNJ10/KCNJ16 (J10/J16, black bar) and KCNJ10/KCNJ16^A188S^ (J10/A188S, red bars) transfected cells showed hyperpolarized membrane potentials close to the equilibrium potential for K^+^ (approximately −90 mV). Membrane potential of KCNJ10/KCNJ16^R137S^ (blue bar) transfected HEK293T cells were depolarized, like the non-transfected cells (gray bar). Data are presented as mean values ± SEM, numbers indicate the numbers of experiments. **c** Whole-cell current of HEK293T clamped at −30 mV: KCNJ10/KCNJ16 (J10/J16, black bar) and KCNJ10/KCNJ16^A188S^ (J10/A188S, red bar) transfected cells showed nearly 4-times higher whole-cell current than KCNJ10/KCNJ16^R137S^ (J10/R137S, blue bar). Data are presented as mean values ± SEM, numbers indicate the numbers of experiments. *(For interpretation of the references to color in this figure, the reader is referred to the online version of this article.)*

Since malfunctioning of potassium channels can be investigated in heterologous expression systems, we decided to take a closer look at the functional properties of the Kir5.1 *(KCNJ16)* variants. Because Kir5.1 does not form functional homomeric channels on its own, Kir5.1 function was analyzed after co-expression with Kir4.1 *(KCNJ10)* in HEK293T cells. Electrophysiological analysis of the two variants of *KCNJ16* revealed that the variant R137S is non-functional, while the variant A188S appears to be fully functional under the experimental conditions used (Fig. 2a–c). This is reflected in the current-voltage curves, which show less than half the whole-cell current in non-transfected (mock) cells and in cells co-transfected with KCNJ10 and the mutant KCNJ16 (R137S) than in cells co-transfected with KCNJ10 and the mutant KCNJ16 (A188S) or WT KCNJ16 (Fig. 2a). In addition, resting membrane potential in HEK293T cells co-transfected with KCNJ10/KCNJ16WT or KCNJ10/KCNJ16^A188S^ showed hyperpolarized membrane potentials close to equilibrium potential for K^+^ (approximately −90 mV), whereas the membrane potential of KCNJ10/KCNJ16^R^^1^^3^^7^^S^ transfected HEK293T cells were depolarized, like the non-transfected cells (Fig. 2b). This was also evident in a whole-cell current that was nearly 4-fold higher in HEK293T cells transfected with KCNJ10/KCNJ16WT or KCNJ10/KCNJ16^A188S^ than in HEK293T cells transfected with  KCNJ10/KCNJ16^R137S^ (Fig. 2c).

## Discussion

One interesting pathway in the pathogenic mechanism of SIDS is the respiratory control system. A number of variants in genes involved in central and peripheral chemoreceptors have already been investigated in SIDS cases.^26,31^ Here, we used whole-exome sequencing and a candidate gene approach to investigate the possible contribution of 11 genes known to influence respiratory control or chemoreception, respectively.

### SIDS cases with potentially impaired respiratory chemoreception

The median age at death of the 5 infants with potentially pathogenic variants in genes of respiratory chemoreceptors was lower compared to the median age at death in SIDS cases with potential cardiovascular genetic diseases.^11,40^ Furthermore, 80% of the SIDS cases with variants in genes involved in chemoreception were male. In most SIDS studies, the male sex accounts for 60% of incidents and is therefore suggested to be an important endogenous risk factor for SIDS.^41^ It has been suggested that males have a more unstable respiratory control compared to females in the postnatal period.^42^ Female sex hormones or an X-linked factor are hypothesized to be responsible for the protective sex-effect in respiratory stability under pathological circumstances and consequentially the lower rate of SIDS in female infants.^41,43^ The early age of death and the predominant male sex in the 5 SIDS victims may indirectly suggest that impaired respiratory chemoreception and thereby compromised respiratory control contributed to these deaths.

### Variants in genes encoding for proteins involved in peripheral chemoreception

In our SIDS cohort, potentially disease-causing variants were found in 2 out of 5 investigated genes encoding for proteins involved in peripheral chemoreception (*OR51E2*, *KCNMA1*).

In a 2-months-old SIDS case, we found a variant (rs777767053, V179F) of uncertain significance in the human olfactory receptor encoding gene *OR51E2*, which might had impaired the CB development or oxygen-sensing. In mice, an age-dependent expression of the olfactory receptor (Olfr78) in the CB and its importance for acute oxygen-sensing has been demonstrated.^44^ However, a more recent study questioned the importance of Olfr78 in oxygen-sensing.^45^ To our best knowledge, the expression of an olfactory receptor subtype in the human CB has not been demonstrated thus far. On the other hand, there is evidence that olfactory receptors in non-olfactory tissues might play a role in chemoreception, lung function, and even SIDS.^46,47^

In a 3-months-old SIDS case, we found a new sequence alteration (S1173G) of uncertain significance in *KCNMA1*. The role of calcium-activated potassium channels (maxi-K^+^ channel; gene *KCNMA1*) in oxygen-sensing in the CB is still not fully understood but these channels seem to be required for the spiking behavior of chemo-sensitive cells in the CB under acute hypoxia, development of the CB and adaption to high altitude.^48^ This new variant might have interfered with CB development and/or acute hypoxia-sensing and thereby could have co-contributed to the death of the infant. Nonetheless, like all potassium channels, maxi-K^+^ is expressed in many other organs, such as the brain, and variants in *KCNMA1* are known to cause epilepsy.^49^ However, sudden unexpected death in epilepsy (SUDEP) is more frequently seen in older children (10.2 ± 5.7 years) and only 2% of all SUDEP appeared in infants under one year of age.^50^

TASK-1 and TASK-3 potassium channel play an important role in oxygen-sensing of the CB.^19^ Moreover, the ability of the CB to measure oxygen depends on mitochondrial complex I (*NDUFS2*).^20^ No potentially pathogenic variants were found in the *TASK-1*,^43^ *TASK-3*, or *NDUFS2* genes in our SIDS cohort. Therefore, genetic variants in the exons of *TASK-1*, *TASK-3* and *NDUFS2* genes seem not to contribute significantly to the cause of death in SIDS.

### Variants in genes encoding for proteins involved in central chemoreception

In our SIDS cohort, we found potentially disease-causing variants in 2 out of 6 investigated genes encoding for proteins involved in central chemoreception *(PHOX2B, KCNJ16*).

It is still under debate whether variants in *PHOX2B* play a role in SIDS.^26,27^ We could not detect the classical CCHS-causing *PHOX2B* polyalanine repeat in our SIDS cohort, but in one SIDS case we found a sequence variant (A118V) in the *PHOX2B* gene, which was not previously reported in the literature or in public databases. In another study, 12 (55%) out of 22 SIDS cases with normal *PHOX2B* polyalanine repeat showed a decreased amount of *PHOX2B* positive stained neurons in the RTN compared to age-matched controls.^51^ The authors hypothesized that some SIDS cases might be explained by unknown *PHOX2B* mutations or mutations in the *PHOX2B* pathway. In CCHS, the absence of RTN neurons and thereby diminished CO_2_ chemoreflex induces apnea during sleep, pointing to the extraordinary importance of this particular chemoreflex for the respiratory drive and arousal during sleep.^25^ SIDS usually occurs during sleep and thus shares an important clinical feature with CCHS. A restricted central CO_2_ chemoreception could therefore be one important reason for the suggested impaired CO_2_ arousal in SIDS during sleep.^52^

Another important central CO_2_ sensor is the locus coeruleus (LC) in the brainstem. Interestingly, the LC is often underdeveloped in the brainstem of SIDS cases.^53^ Inwardly rectifying potassium channel 5.1 (Kir5.1/*KCNJ16*) is suggested to be important for the ability of the LC to measure CO_2_.^30^ In addition, Kir5.1 appears to be essential for the acute and chronic regulation of arterial pH through its key function of renal H^+^ absorption / ^+^ secretion and is thus an important determinant of the ventilatory CO_2_ chemoreflex.^54^ We found in two SIDS cases potentially pathogenic exonic SNPs in *KCNJ16* (R137S and A188S), which may interfere with central CO_2_ sensitivity and thereby have contributed to the cause of death. In electrophysiologic analysis, however, only the R137S *(rs766250689)* was impaired, whereas the variation A188S (new variant) was fully functional in the expression system used and when co-expressed with Kir4.1. Recent research suggests that Kir5.1 may also form functional heterodimers with other K^+^ channels e.g., Kir4.2 (*KCNJ15*). It is therefore conceivable that the SNP A188S found in this study could affect the function of Kir5.1 co-expressed with other K^+^ channels, such as Kir4.2. The SNP R137S, however, is located in the genetic region encoding for pore formation and its’ negative effect on Kir5.1 function should therefore not be dependent on the co-expressed K^+^ channel.

We did not find potentially pathogenic variants in other transcription factors (*ATOH1*, *MECP2*), which are known to interfere with neuronal development of putative central CO_2_ chemo-sensitive neurons in our SIDS cohort. In addition, no variants were found in *KCNK5* (TASK-2) and *GPR4* which are essential for the ability of *PHOX2B* positive neurons to measure CO_2_-changes in the brainstem.^28^

### Key proteins of respiratory chemoreception

Genetic variants in the exons of key genes encoding for proteins of respiratory chemoreception may play a role in only 3% of our SIDS cases, despite impaired or immature respiratory chemoreception previously suggested to be an important risk factor for SIDS.^17^ A number of considerations may account for this. First of all, many other proteins which are known to be involved in respiratory chemoreception were not investigated in this study. In this regard, genetic investigation of more genes involved in respiratory chemoreception might have disclosed more potentially pathogenic variants in our SIDS cases, such as in studies investigating cardiac diseases.^11,40^ This would necessarily make the interpretation of these results increasingly more complex given how many proteins (e.g., potassium channels) are expressed across organ systems with different functions and are causative for various diseases when their function is impaired.^39,55–59^ Furthermore, some genes known to be involved in respiratory chemoreception have already been investigated in the context of cardiac diseases and have therefore been excluded from this study.^11,40^ The number of SNPs in a gene may depend on various factors, such as the length of the gene and ethnicity. It is further assumed that SNPs are less frequent in genes encoding for proteins with a probability of being loss-of-function intolerant (pLI ≥ 0.9).^60^ In this study, 4 of the 11 genes examined here had a pLI ≥ 0.9, which may have contributed to the fact that we found rare variants (MAF < 0.001) in only 5 SIDS cases. One case–control study found in 405 genes ultra-rare variants (MAF < 0.00005) in an European SIDS cohort (*n* = 247), but not in the 11 genes that we investigated.^61^ It is therefore possible that more or less variants in the 11 genes studied here could be found in other SIDS cohorts. Increased collaboration and expansion of SIDS cohorts in the future may help to better understand the possible role of these 11 genes in SIDS.^5^

### Impact of genetic variants

It is very challenging to interpret the impact of genetic variants on protein and organ function and to subsequently characterize the genotype/phenotype relationship.^37,62^ It is important that the categorization of variants is based on a stringent scoring system^37^, such as those we have successfully applied in previous studies.^11,63^ Unfortunately, these scoring systems are never as powerful as functional studies. For all 5 variants described in our study, we did not find any functional studies in the literature. For the two identified variants in *KCNJ16* (Kir5.1), an electrophysiological analysis was performed, in which the R137S variant showed a loss-of-function.

A challenge for interpreting the findings is the fact that all variants identified in this study were heterozygous. At present, it is unclear if these variants have pathogenic effects in the specialized cells controlling respiration or if the possibly reduced quantity of the normal gene product is insufficient for a normal phenotype (haploinsufficiency). Unfortunately, it is difficult to mimic the heterozygous state and haploinsufficiency in an artificial expression system because minor changes may only be visible in the context of native cells.

Introns are not covered in whole-exome data and we could not examine the impact of intronic SNPs in the investigated genes. In genome-wide association studies (GWAS), most SNPs are found in non-coding regions, and their impact on the development of various diseases is still under debate.^64^ Intronic SNPs are suggested to cause changes in gene expression levels rather than causing changes to protein function, which could have an effect on the development of chemoreceptive organs in SIDS cases.^53,64^ The untranslated gene regions (5′- and 3′UTR) contain numerous regulatory elements like CpG sites, upstream open reading frames, and RNA binding sites. Genetic variants modifying these regions can have an important impact on the overall production of the protein by affecting RNA transcription, stability, and translation.^65^ Besides, deep intronic variants can disrupt transcription regulatory motifs or inactivate intron-encoding genes.^66^ Therefore, the analysis of the here investigated 11 genes should not only include rare pathogenic variants but also non-coding variants, which could in association with other variants contribute to an increased susceptibility of life-threatening events.^67^

Moreover, the determination of the mode of inheritance to classify variants into the pathogenic category by co-segregation of family members was not possible in our study, due to the anonymization required by the ethical committee.

## Conclusion

In summary, missense variants in genes involved in respiratory chemoreception may play a role in a minority of SIDS cases. This is in contrast to other investigated pathophysiological pathways, such as lethal cardiac diseases, where up to 30% of SIDS cases were reported to be affected by likely pathogenic variants. Most likely variants in central and peripheral chemoreception are not the only cause of death, but may contribute to the multifactorial etiology of SIDS. Functional and more extensive genetic studies are required to prove this hypothesis.

## Supplementary Information

Supplementary data
