# Supplementary material for: Genetic variants in eleven central and peripheral chemoreceptor genes in sudden infant death syndrome
Source: Pediatr Res. 2022 Feb 1;92(4):1026–33. doi: 10.1038/s41390-021-01899-4 (PMC9586864; doi:10.1038/s41390-021-01899-4)
Supplement: Supplementary file 1 — Supplementary data [file 41390_2021_1899_MOESM1_ESM.pdf]

# Supplementary data

**Table S1** Detailed information on the 11 investigated genes.

| Gene | Chr. | Start position | End position | Length | Nr. of exons | Coding sequence length (nt) | Protein length (aa) |
|---|---|---|---|---|---|---|---|
| *ATOH1* | 4 | 94'750'042 | 94'751'221 | 1'179 | 1 | 927 | 309 |
| *GPR4* | 19 | 46'093'022 | 46'105'466 | 12'444 | 2 | 1'086 | 362 |
| *KCNJ16* | 17 | 68'049'570 | 68'131'749 | 82'179 | 5 | 1'254 | 418 |
| *KCNK3* | 2 | 26'915'619 | 26'956'288 | 40'669 | 2 | 1'185 | 394 |
| *KCNK5* | 6 | 39'156'749 | 39'197'226 | 40'477 | 5 | 1'500 | 499 |
| *KCNK9* | 8 | 140'613'081 | 140'715'299 | 102'218 | 4 | 1'125 | 374 |
| *KCNMA1* | 10 | 78'629'359 | 79'398'353 | 768'994 | 28 | 3'711 | 1'236 |
| *MECP2* | X | 153'287'024 | 153'336'212 | 49'188 | 4 | 1'497 | 498 |
| *NDUFS2* | 1 | 161'166'894 | 161'184'185 | 17'291 | 13 | 1'392 | 463 |
| *OR51E2* | 11 | 4'701'401 | 4'719'084 | 17'683 | 2 | 960 | 320 |
| *PHOX2B* | 4 | 41'746'099 | 41'750'987 | 4'888 | 3 | 416 | 139 |

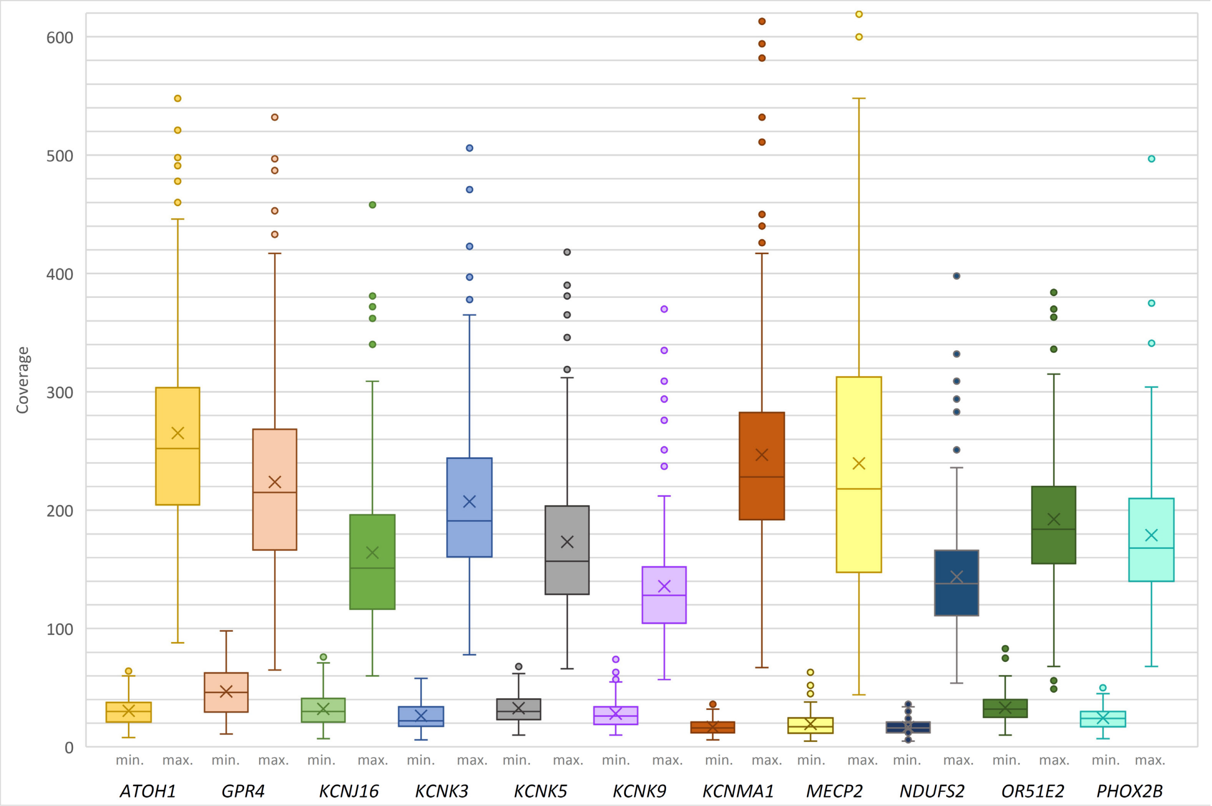


**Figure S1** Minimum and maximum coverage of the exonic part of the 11 genes investigated in the SIDS cohort. Within the 11 genes, the minimum mean exonic coverage was 29.98 ± 15.01 and the maximum mean exonic coverage was 197.39 ± 87.13.

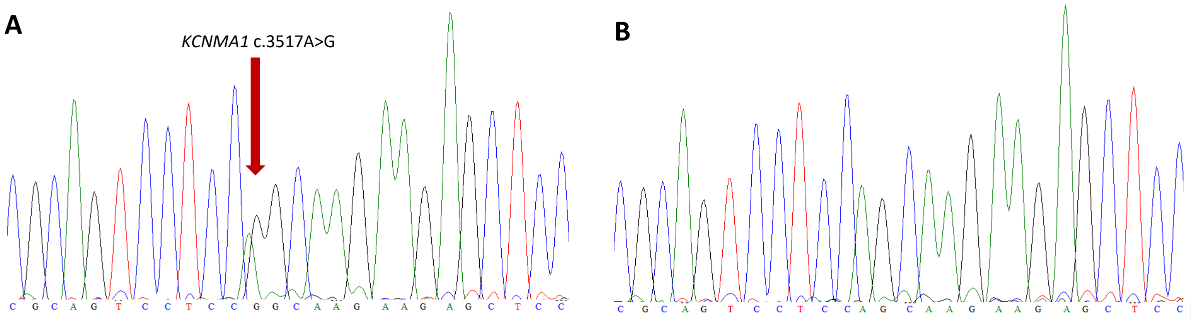


**Figure S2** Sanger sequencing confirmation of the identified variant in the gene *KCNMA1* (NM_001322837.1; c.3517A>G) in A) SIDS192 and in B) a control sample. The sequencing was performed with the BigDye$^{TM}$ Terminator v3.1 cycle sequencing kit (ThermoFisher Scientific, Reinach, Switzerland) according to manufacturer's instruction. The software BioEdit Sequence Alignment Editor version 7.1.3.0 (https://bioedit.software.informer.com/7.2/) was used for visualization. Primer sequences are available on request.
